# Impact of Inherited Genetic Variants on Critically Ill Septic Children

**DOI:** 10.3390/pathogens11010096

**Published:** 2022-01-14

**Authors:** Mariana Miranda, Simon Nadel

**Affiliations:** 1 Paediatric Unit, Imperial College Healthcare NHS Trust, London W2 1NY, UK; 2 St. Mary’s Hospital, Imperial College Healthcare NHS Trust, and Imperial College, London W2 1NY, UK; simon.nadel@nhs.net

**Keywords:** children, epigenetics, gene association studies, genome-wide expression profiling, host-pathogen response, immunity, inherited genetic variants, phenotypes, sepsis

## Abstract

Sepsis remains an important source of morbidity and mortality in children, despite the development of standardized care. In the last decades, there has been an increased interest in genetic and genomic approaches to early recognition and development of treatments to manipulate the host inflammatory response. This review will present a summary of the normal host response to infection and progression to sepsis, followed by highlighting studies with a focus on gene association studies, epigenetics, and genome-wide expression profiling. The susceptibility (or outcome) of sepsis in children has been associated with several polymorphisms of genes broadly involved in inflammation, immunity, and coagulation. More recently, gene expression profiling has been focused on identifying novel biomarkers, pathways and therapeutic targets, and gene expression-based subclassification. Knowledge of a patient’s individual genotype may, in the not-too-remote future, be used to guide tailored treatment for sepsis. However, at present, the impact of genomics remains far from the bedside of critically ill children.

## 1. Introduction

The unpredictability of response to infection among individuals is one of the most notable characteristics of infectious diseases. The questions include—why do some organisms, which usually behave as harmless commensals, being carried intermittently or throughout life, cause invasive disease in otherwise healthy children? Why can organisms responsible for infection in children, who are able to control and eradicate these organisms, trigger severe infection in others, who, with the same appropriateness of antimicrobial therapy and organ support, die of overwhelming infection or of over-exuberant inflammatory response? The answer appears to be multifactorial, but is determined to some degree, in our genetically programmed response to infection [1].

Sepsis is a clinical syndrome caused by a dysregulated host response to severe infection. The normal host response to infection is a complex process that focuses on localizing and controlling the bacterial invasion, followed by repair of the injured tissue. In this process, there is activation of circulating and fixed phagocytic cells, alongside with generation of proinflammatory and anti-inflammatory mediators secreted by macrophages, which have been triggered and activated by the bacterial invasion of tissue [2].

Sepsis continues to be an important source of morbidity and mortality in children, despite the development of standardized treatment guidelines, vaccines, and intensive care organ support techniques. The cause of why some infections spread beyond the local environment causing sepsis is likely multifactorial. It may include the direct effects of the invading microorganisms or their toxic products, release of large quantities of proinflammatory mediators, complement activation, and host factors, as some individuals may be genetically susceptible to developing sepsis [1,2].

Determining why certain infections continue to have a high mortality in specific groups of patients may provide clues to novel therapeutic interventions. There has been limited success with treatments to manipulate the inflammatory response, undoubtedly due to limited understanding of the complex mechanisms, which regulate the innate immune response. However, research confirms that sepsis is a heterogeneous syndrome, within which there probably exist several biological subclasses. Therefore, the design of more specifically targeted therapies should consider the potential influence of ethnic and genetic diversity [2,3].

In the last decades, there has been an increased interest in genetic and genomic approaches to early recognition of sepsis, and in the development of treatments to manipulate the inflammatory response in sepsis [3].

## 2. Normal Host Response to Infection

The recognition and binding to microbial components by innate immune cells, particularly by activated macrophages, marks the initiation of the host response to an infection. This process can occur by several pathways:

- **Pattern recognition receptors (PRRs) on the surface of host immune cells can recognize and bind to the pathogen-associated molecular patterns of microorganisms** [2]. PRRs include toll-like receptors (TLRs), nucleotide-oligomerization domain (NOD) leucine-rich repeat proteins, and retinoic-acid-inducible gene I (RIG-I)-like helicases. Examples include peptidoglycan of Gram-positive bacteria binding to TLR-2 on host immune cells, lipopolysaccharide of Gram-negative bacteria binding to TLR-4, and lipopolysaccharide-binding protein (CD14 complex) on host immune cells. **PRRs can also recognize endogenous danger signals, also known as danger-associated molecular patterns that are released during the inflammatory process**. These are nuclear, cytoplasmic, or mitochondria structures, acquiring new functions when released in the extracellular environment. Examples include high mobility group box-1 protein HMGB1, S100 proteins, heat shock proteins, mitochondrial DNA and metabolic molecules, such as ATP [4];
- Triggering **myeloid cell (TREM-1) and myeloid DAP12-associating lectin (MDL-1) receptors** on host immune cells that recognize and bind to microbial components [5];
- **Other cell structures released during infection, including microparticles from circulating, vascular cells, and formation of neutrophil extracellular traps (NETs)** also contribute to the deleterious effects of sepsis-induced intravascular inflammation. Although the trapping strategy of NETs is important to immobilize and kill invading microorganisms, NET release of nuclear chromatin (DNA, histones) and bactericidal proteins can promote inflammatory host response, endothelial damage, and thrombosis [6,7].

After recognition, the binding of immune cell surface receptors to microbial components is responsible for the activation of several processes, including:

- **Toll-like receptors (TLRs) are going to trigger a signaling cascade, via the activation of cytosolic nuclear factor-kb (NF-kb)**—the activated NF-kb will bind to transcription sites, inducing activation of a large set of genes involved in the host inflammatory response, such as **proinflammatory cytokines** (tumor necrosis factor alpha [TNFα], interleukin-1 [IL-1]), **chemokines** (intercellular adhesion molecule-1 [ICAM-1], vascular cell adhesion molecule-1 [VCAM-1]), and nitric oxide [2];
- **Activated polymorphonuclear leukocytes (PMNs) will express adhesion molecules that cause their aggregation and margination to the vascular endothelium**—this process will be accelerated by the endothelium expressing adherence molecules to attract leukocytes, followed by a series of steps (rolling, adhesion, diapedesis, and chemotaxis) to migrate to the injury site. PMNs will then release mediators locally, responsible for the basic signs of inflammation: warmth and erythema due to local vasodilation and hyperemia, and protein-rich edema due to increased microvascular permeability [2];
- **Complement activation**—is a proinflammatory and membrane-altering host defense system that is part of both innate and adaptive immunity, also playing a role in removal of damaged cells, tissue regeneration, and angiogenesis. It consists of plasma proteins that may be divided into three major cascades: the classical, lectin, and alternative pathways. Although each pathway is activated by a different set of situations, all result in the formation of a proinflammatory environment, deposition of large amounts of C3 on the target cell (leading to opsonization), and membrane lysis by the membrane attack complex (MAC) [8,9].

A combination of proinflammatory and anti-inflammatory mediators, secreted by activated macrophages, which will then regulate this host inflammatory response, are described in Table 1.

The release of TNFα is self-sustaining in sepsis due to autocrine secretion, as the binding of endotoxin to lipopolysaccharide (LPS)-binding protein and its subsequent transfer to CD14 on macrophages will stimulate TNFα release [13]. While non-TNF cytokines and mediators (e.g., Il-1, IL-2, IL-6, IL-8, IL-10, platelet activating factor, interferon, and eicosanoids) act by increasing the levels of other mediators. This proinflammatory environment leads to the recruitment of more PMNs and macrophages, creating a self-sustaining cycle.

The regulation of the inflammatory processes, including adherence, chemotaxis, phagocytosis of invading bacteria, bacterial damaging, and phagocytosis of debris from injured tissue, is going to depend on the equilibrium of proinflammatory and anti-inflammatory mediators.

## 3. Progression to Sepsis

Although inflammation is an essential host response to any infection, the progression to a dysregulation of the normal response causes the activation of a chain of events that leads to widespread tissue injury, immune and microcirculatory dysregulation, characteristic of systemic inflammatory response syndrome (SIRS). It is this uncontrolled, unregulated, and self-sustaining intravascular inflammation, rather than the primary infectious microorganism, that leads to end-organ dysfunction in tissues remote from the original insult, eventually causing multiple organ failure [2,12].

It is unclear why immune responses that generally remain localized sometimes spread beyond the local environment, causing sepsis. The reason is probably multifactorial and may include the direct effects of the invading microorganisms, their toxic products, release of proinflammatory mediators, complement activation and, in some individuals, genetic susceptibility to developing sepsis might be significant.

Previous studies in adults also report a state of acquired innate and adaptive immune suppression or “immune paralysis” in some patients, simultaneously or after the early proinflammatory response [14,15,16,17].

## 4. Genetic Susceptibility

The classic example of a clear link between genetics and immune dysfunction are the single-gene Mendelian mutations causing primary immunodeficiencies, such as immunoglobulin deficiency, complement deficiencies, T- and B-cell deficiency, and neutrophil and innate immune defects. These are rare and mostly present early in life with severe or recurrent infections with multiple different pathogens. Nevertheless, most critically ill children do not have a primary immunodeficiency and studies in adoptees showed that an individual’s genetic heritage impacts the outcome from infection [3,18]. Data from the Human Genome and the HapMap projects suggest that common genetic variants might contribute to an individual’s susceptibility to infection, disease severity, and/or response to treatment.

The role of genetic variants in pediatric sepsis is mostly explored using gene association studies (using a candidate gene for proteins known to be involved in the disease), as genome wide association studies (GWAS) may require hundreds to thousands of patients and collecting these cohorts of children is difficult. In the last few years, most research has been focusing on single nucleotide polymorphism (SNP), sites in the DNA sequence where individuals differ at a single DNA base due to stable substitution, deletion, or insertion of a single nucleotide. Although they are the most common form of genetic variation, with a frequency of more than 1% in at least one population and are scattered throughout the genome, only 2 to 3% of SNPs alter the function/expression of a gene, thus resulting in an altered protein or variation in the amount of normal protein expression.

A set of nearby SNPs on the same chromosome is called a haplotype and they are inherited in blocks. Blocks may contain a large number of SNPs, but a few SNPs are enough to uniquely identify the haplotypes in a block. The HapMap is a map of these haplotype blocks and the specific SNPs that identify the haplotypes are called tag SNPs. This is a powerful resource for studying the genetic factors contributing to variation in response/susceptibility to infection, by reducing the number of SNPs required to examine the entire genome for association with a phenotype [3,19].

Several polymorphisms of genes broadly involved in inflammation, immunity, and coagulation have been associated with susceptibility, or outcome of sepsis in children. SNPs associated with increased susceptibility to infection and/or poor outcomes include SNPs of genes that encode cell surface receptors (e.g., CD14, MD2, toll-like receptors 2 and 4, and Fc-gamma receptors II and III), cytokines (e.g., TNF, lymphotoxin-alpha, IL-10, IL-18, IL-1 receptor antagonist, IL-6, and interferon gamma), lipopolysaccharide ligands (LPS binding protein, bactericidal permeability increasing protein), angiotensin I-converting enzyme, plasminogen activator inhibitor, mannose-binding lectin, heat shock protein 70, and caspase-12 [3,20,21,22,23].

However, it is important to note that a genotype that has a protective role in prevention infection could be deleterious if systemic infection occurs. For example, studies suggest that although increased TNFα response decreases the risk of infection, it can increase the risk of death from septic shock, should it occur. Therefore, the sequelae of an altered host response may have competing effects in population-based studies [3]. Table 2 summarizes some of the more relevant associations studied to date.

It should be noted that not all of these associations have been confirmed independently. There are rigorous criteria determining the quality of an ideal gene association study and, unfortunately, many gene association studies do not meet this level of rigor and the results may not be applicable to all populations [36,37].

Many of these studies involve meningococcemia and the impact of PAI-1 4G allele on outcome in meningococcal disease is perhaps the most established association between genetic variation and outcome in pediatric septic shock. Although, at present, no gene association study has directly been translated to the bedside in the form of a novel therapy, the concept is still valid and positive association studies will need to be validated, focusing on functional polymorphisms with reasonable therapeutic options [3,24,25].

## 5. Epigenetic Process

The complexity of the inflammatory response begins with the control of expression of inflammatory genes. Epigenetic mechanisms are key heritable regulators in gene expression that are not related to direct DNA sequence changes. However, they can regulate inflammatory gene expression and appear to have implications on inflammatory responses in critically ill children [3,38]. Epigenetic mechanisms include DNA methylation, non-coding RNA (microRNA) silencing of gene expression, and histone post-translational modifications [38,39].

The microRNAs (miRNAs) have been increasingly recognized as dynamic regulators of various transcriptional and signaling pathways, making it plausible that miRNAs may be potential biomarkers of various phases of sepsis and feasible targets for therapeutic intervention in pediatric sepsis. MicroRNA levels can be evaluated in different body fluids, such as blood, serum, plasma, and blood cultures expanding the range of options for epigenetic studies. Multiple studies showed that miRNAs are involved in the regulation of the exacerbated inflammation, endothelial dysfunction, and coagulation cascade in sepsis, including the targets described in the Table 3 [40,41,42].

Furthermore, miRNAs could play a critical role as new biomarker for early diagnosis and early prognosis of sepsis. For example, studies showed that miR-16, miR-122, and miR-133a are increased in serum of septic patients, whereas the expression of some miRNAs, such as miR-25 and miR-181b is decreased [41,42]. In addition, a recent study reported that the decrease in level of miR-25 was correlated with the severity of sepsis, with better clinical accuracy for sepsis diagnosis than CRP and PCT levels [42,53].

Once again, we should not ignore the fact that the significance of the findings is influenced by the precise patient population studied and the methods used. Large-scale clinical trials are essential to successfully translate these into clinical practice.

Lastly, in children with septic shock, there is evidence of differential expression of genes involved in epigenetic regulation, inducing epigenetic changes in dendritic cells and lymphocytes causing a state of immune-deficiency for a prolonged period after the initial sepsis challenge, in parallel with suppression of adaptive immunity genes. This might explain why some studies show that patients who recover from severe sepsis are at increased risk of death for several years after discharge [3,18,38,39].

## 6. Expression Profiling Studies

Gene expression profiling is a whole-genome approach that involves the measurement of activity (and so the expression) of thousands of genes at once, using microarray technology to simultaneously measure mRNA abundance of thousands of transcripts, creating a global picture of cellular function [3,53]. These profiles can, for example, show how cells react to a particular treatment.

It is a hypothesis-generating approach, rather than hypothesis-driven, as no a priori assumptions are made concerning the relevance of any genes to the biological process being studied. The ability to cross-examine the entire genome provides an opportunity to illuminate complex pathogen recognition and inflammatory signaling pathways of previously unrecognized targets relevant to sepsis biology. More recently, gene expression profiling has been focused on identifying novel biomarkers, pathways, therapeutic targets, and gene expression-based subclassification [3,54]. The Table 4 describes some of these studies.

## 7. Conclusions

Pediatric sepsis is characterized by a high complexity of pathophysiological and molecular mechanisms. The prospect of enhancing our understanding of the genetic contribution to the susceptibility, host response, outcome of sepsis, and the role of epigenetics in influencing gene expression in critically ill children remains one of the most exciting areas of sepsis research.

Useful biomarkers that might be able to reduce the need for genome-wide approaches in the future, thus potentially allowing near patient testing and enabling the translation of gene expression research into clinical practice, are in development. Knowledge of a patient’s individual genotype may shortly be used to guide individual patient-tailored treatment for sepsis. Recent evidence suggests that epigenetic regulation may play a central role in the pathogenesis of sepsis, uncovering exciting prospects for potential novel diagnostic and therapeutic approaches.

However, the impact of genome science currently remains far from the bedside of critically ill children. The introduction of such an approach into routine clinical practice will have to await the results of further rigorous studies, in larger, more homogeneous cohorts of patients, with an emphasis on independent validation.

## Figures and Tables

**Table 1:** Proinflammatory and anti-inflammatory mediators [10,11,12].

Proinflammatory Mediators	Anti-Inflammatory Mediators
Like **TNFα and IL-1**, are responsible for an important range of biological effects: -Fever -Hypotension -Acute phase protein response -Production of IL-6 and IL-8 -Coagulation activation -Fibrinolytic activation -Leukocytosis -Stress hormone response -Enhanced endothelial permeability (TNFα) -Neutrophil degranulation and enhanced antigen expression (TNFα) -Increased gluconeogenesis (TNFα) -Increased lipolysis (TNFα)	**Cytokines that suppress the immune system by inhibiting the production of TNFα and IL-1** by mononuclear cells and monocyte-dependent T helper cells. However, their effects may not always be anti-inflammatory. As examples, IL-10 and IL-6 both enhance B cell function (proliferation and immunoglobulin secretion) and encourage the development of cytotoxic T cells.

**Table 2:** Summary of relevant genetic susceptibility associations studied published to date.

Host Factor	Clinical Effect
**Genetic Variation in Genes Involved in the Host Response to Pathogens**	
**Tumor-necrosis factor-α(TNFα)**	TNF2 allele and G308A mutation in TNF promoter associated with increased serum levels of TNFα, linked increased susceptibility to septic shock and illness severity in meningococcemia [3,24,25,26,27].
**Lymphotoxin-α (TNF-β)**	TNFβ2 allele in bacteremia children associated with higher systemic levels of TNFα and higher mortality [3,25,26].
**Interleukin-1 (IL-1) and IL-1 receptor antagonist (IL-1RA)**	IL-1B (−511) alleles associated with increased survival in meningococcemia. Combination of the IL-1B (−511) and IL-1RN (+2018) alleles associated with decreased survival. Further, IL1RA (+2018) polymorphism was associated with risk of meningococcal disease and worse outcome [3,21].
**Interleukin-6 (IL-6)**	G-174C and G-572C polymorphisms could be predictors of risk of development and/or predictors of sepsis severity, however conflicting findings in some studies [3,21].
**Genetic variation in pathogen recognition molecules**	
**Toll-like receptor 4 (TLR4)**	Asp299Gly allele led to abnormal responses to endotoxin, with significant association with Gram-negative sepsis. TLR4 polymorphisms have been linked with susceptibility to malaria and meningococcal disease in children [3,28,29].
**Toll-like receptor 2 (TLR2)**	Arg753Gln allele renders TLR2 less responsive to components of Gram-positive bacteria, although subsequent studies have not been able to confirm a strong association with severity of Gram-positive sepsis [3,28,30,31].
**Adapter protein Mal (TIRAP)**	Adapter proteins are the downstream signaling apparatus of TLRs. Polymorphisms in TIRAP have been linked to invasive pneumococcal disease and susceptibility to invasive *Haemophilus influenzae* infection in immunized children [3,32,33].
**Mannose binding lectin (MBL)**	Polymorphisms associated with increased susceptibility to meningococcal disease, pneumonia and sepsis in neonates, severe infections, and increased risk of acute respiratory infections in children 6 to 17 months of age [3,21].
**Effector molecules involved in the immune response**	
**Fc gamma receptor**	Polymorphisms associated with increased susceptibility to infections by encapsulated bacteria and illness severity, although conflicting findings in some studies [3,21].
**Bactericidal permeability** **increasing protein (BPI)**	Polymorphisms associated with increased risk of Gram-negative sepsis [3,21].
**Surfactant protein A2**	Polymorphisms associated with increased illness severity in infants with RSV infection [3,21].
**Complement factor H**	Polymorphisms associated with increased risk of invasive meningococcal disease, increased serum factor H levels, and reduced bactericidal activity against meningococcus [3,21].
**Angiotensin converting** **enzyme (ACE)**	DD genotype associated with increased illness severity in meningococcal disease [3,21].
**NOD2 receptor**	Gene variants of the NOD2 receptor (pathogen recognition receptor) associated with increased risk of sepsis and illness severity [3,21].
**Genetic variation in the host coagulation system**	
**Plasminogen-activator** **inhibitor 1 (PAI-1)**	4G/4G haplotype polymorphism causes increased levels of PAI-1 (pro-coagulant factor as it inhibits fibrinolysis), associated with increased severity and mortality in meningococcal disease [3,34,35].
**Protein C promoter**	Carriers of C-1654T and A-1641G allele present increased risk of developing meningococcal sepsis. Low plasma protein C levels have been correlated with increased severity of illness and poor outcome in meningococcal sepsis [3,21].
**Fibrinogen**	Fibrinogen and fibrinogen degradation products are potent chemo-attractants and induce release of IL-8 from neutrophils. Carriage of the factor XIII-Val34Leu polymorphism was associated with a higher rate of sepsis among very low birth newborns of mixed European descent [21].

**Table 3:** Summary of the more relevant miRNAs targets in pediatric sepsis published studies to date.

miRNA Target	Clinical Effect
**miR-223**	Pro-inflammatory factor involved in several signaling pathways that control inflammatory responses, such as modulating the differentiation of neutrophils and macrophages, negative regulation of STAT3, IL-6, IL-1β and TNFα production, by the TLR4/TLR2/NF-κB pathway during sepsis. Higher levels detected in severe sepsis [40,43].
**miR-146a, mi-R125, mi-R155**	Critical role in the negative regulation of TLR/NF-κB mediated innate immune and inflammatory responses. Serum miR-146a levels were significantly decreased in pediatric septic patients. Serum miR-146a expression was negatively associated with protein c-reactive (PCR), pro-calcitonin (PCT), IL-6, and TNFα, which can reflect the severity [40,44]. Furthermore, high levels of miR-155 in septic adult patients positively correlated with a higher sepsis-related organ failure assessment score (SOFA) and greater severity of sepsis [40,45].
**miR-15a, miR-16a, miR-125b, miR-146a**	Shown to prevent NF-κB activation in sepsis. Upregulation of miR-125b correlated with disease severity, inflammation, and increased mortality in adult sepsis and positively correlated with other markers of sepsis, such as CRP, PCT, TNFα, and IL-6 levels [40,46].
**miR-26a**	Reported to reduce inflammatory responses possibly by enhancing regulatory T-cell responses or by inhibiting NF-kB. Downregulation negatively correlated with IL-6 expression in neonatal sepsis [40,47].
**miR-23b**	Negatively regulates the inflammatory responses induced by LPS, targeting matrix metalloproteinase 10 (MMP-10). Moreover, an essential contributor to the activation of cardiac fibrosis in late sepsis [40,48]. miR-23b levels have been shown to be markedly downregulated in blood cultures of newborns who died from sepsis [40,49].
**miR-101**	miR-101 expression is influenced by virus infection and proinflammatory cytokines. Positively correlated with serum IL-6 and TNFα levels. Can be a negative regulator in macrophages via MAPK phosphatase-1 [40,50].
**miR-130**	NF-κB/DICER (a member of the ribonuclease (RNase) III family) signaling through the generation of mature forms of miR-130a can suppress TNFα expression [40,51]. miR-130a expression is downregulated in severe septic patients with thrombocytopenia, and a study suggested that direct neutralization of proinflammatory IL-18 using miR-130a (which indirectly inhibits IL-18), may be a promising approach for treating severe sepsis in adult patients with thrombocytopenia [40,52].

**Table 4:** Summary of relevant Gene expression profiling studies published to date.

Factor	Clinical Effect
**Discovery of Novel Pathways and Therapeutic Targets**	
**Interleukin-6**	Major contributor to myocardial depression in meningococcal sepsis [3,55].
**Zinc homeostasis**	Early and persistent repression of gene programs directly related to/dependent on zinc homeostasis, as well as low serum zinc concentrations were reported in children with septic shock [3,56]. Correlation between low plasma zinc concentration and higher illness severity [57]. Although, the safety and efficacy of zinc supplementation in clinical sepsis remains to be directly validated.
**Metalloproteinase-8 (MMP-8)**	Highest expressed gene in patients with septic shock and septic shock non-survivors, compared to survivors. Studies showed that pharmacologic inhibition or genetic ablation of MMP-8 grants a significant survival advantage in a murine model of sepsis [3,58].
**Receptor on** **myeloid cells 1** **(TREM-1)**	Triggering TREM-1 is critical for amplification of the inflammatory response to pathogen challenge. A recent study showed that TREM-1 pathway may not be particularly active in neonates with sepsis, illustrating how some candidate therapeutic strategies may not have a biological foundation across all age groups [3,5].
**Discovery of sepsis biomarkers**	
**Bacterial versus** **viral infection** **biomarkers**	Multiple studies have applied gene expression profiling to differentiate bacterial versus viral infection in hospitalized febrile children, including expression signatures that can distinguish: influenza A from bacterial infection; *E. coli* from *S. aureus* infection; children with systemic onset juvenile idiopathic arthritis (as an example of “sterile inflammation”) from children with acute bacterial or viral systemic infections [3,59,60].
**Interlerukin-8** **(IL-8)**	Differentially regulated gene between survivors and non-survivors in children with septic shock. A subsequent study demonstrated that a serum IL-8 level of 220 pg/mL or less, obtained within 24 h of admission, predicts a high likelihood of survival in children with septic shock [61].
**Discovery of gene expression-based septic shock subclasses**	
Studies in pediatric cohorts reported pediatric septic shock subclasses, suggesting that rapid and dynamic shifts in transcription patterns, associated with various phases of sepsis, may account for some of the heterogeneity seen in sepsis [62]. Tree subclasses of (subclasses “A”, “B”, “C”) were identified using a computer algorithm that groups patients based on statistically similar patterns of gene expression, with no a priori knowledge of the clinical phenotype. Post hoc analysis of the clinical subclass phenotypes revealed that subclass A patients had a significantly higher level of illness severity, including mortality [3,63,64]. In a subsequent study, the subclass-defining gene expression patterns were concentrated to a 100-gene expression signature and depicted using visually intuitive gene expression mosaics [3,64,65]. They were measured within the first 24 h of a severe sepsis diagnosis, reflecting gene expression in response to severe sepsis. Once again, the subclass A patients were independently associated with mortality and greater organ failure burden, even after accounting for age, illness severity, and co-morbidity burden [63,64,65]. Because the subclass-defining genes reflect adaptive immunity and glucocorticoid receptor signaling, they have the potential to help in therapeutic decisions. For example, prescription of corticosteroids was independently associated with increased odds of poor outcome among subclass A children [66].	
